# Diabetic Retinopathy in Italy: Epidemiology Data and Telemedicine Screening Programs

**DOI:** 10.1155/2016/3627465

**Published:** 2016-11-21

**Authors:** Stela Vujosevic, Edoardo Midena

**Affiliations:** ^1^Department of Ophthalmology, University of Padova, Padova, Italy; ^2^Fondazione G. B. Bietti, IRCCS, Roma, Italy

## Abstract

In Italy, the number of people living with diabetes is about 3.5 million (5.5% of the population), with an increase by about 60% in the last 20 years and with 1 person out of 3 older than 65 years. The Italian Health Service system estimates that 10 billion euros is spent annually on caring for patients with diabetes, a figure that increases yearly. No national data on prevalence and incidence of legal blindness in patients with diabetes and no national registry of patients with diabetic retinopathy (DR) are currently available. However, the available epidemiological data (in several locations throughout the country) are consistent with those reported in other European countries. The use of telemedicine for the screening of DR in Italy is confined to geographically limited locations. The available data in the literature on implementation and use of telematic screening proved to be successful from patient, caregiver, and authorities point of view. This review addresses the available epidemiological data on DR and telematic screening realities in Italy and thus may help in establishing a national screening program.

## 1. Diabetes Mellitus: The Italian Scenario

Diabetes mellitus (DM) is considered a global epidemic of the 21st century with currently 382 million people affected worldwide and with a projection of doubling this number (592 million) by 2035, as estimated by the World Health Organization [1]. In Italy, the number of patients with DM has increased by about 60% in the last 20 years, from 3.4% in 1993 to 5.5% (thus 3.5 million people) [2–4]. Recent epidemiologic data from the ARNO observatory (a partnership between the Italian Society of Diabetology and the Inter-University Consortium ARNO Cineca) reported that 1 person out of 3 affected by DM is older than 65 years, and of these, 1 out of 4 is older than 75 years of age [2]. Less than 1% are younger than 20 years and 3% are younger than 35 years [2]. The prevalence of DM is 6.1% in men and 5.5.% in women with a consistent difference of 10% across all age groups >35 years [2]. Currently 67% of patients are treated with oral hypoglycemic drugs, 10% of them with a combination with insulin and 11% with insulin alone [2]. It is estimated that patients with type 1 diabetes mellitus (T1DM) represent approximately 2-3%, whereas patients with type 2 diabetes mellitus (T2DM) represent more than 90% of all patients with known DM in Italy [5]. The Bruneck study (long-term, prospective, population-based study in the town of Bruneck located at the very north of Italy) reported an incidence rate of 7.6 per 1,000 person-years of T2DM in individuals aged 40–79 years and independent risk factors for incident DM as follows: impaired fasting glucose, overweight/obesity, insulin resistance, and impaired insulin response to oral glucose [6]. In the province of Torino, the incidence rate of T1DM in the age group of 30–49 years was 7.3 (6.2–8.6) per 100,000 person-years, being at least as high as that in the age group of 15–19 years (6.8, 6.3–7.4) [7]. Men had two-fold higher risk for developing T1DM than women in all age groups [7]. The incidence of known T2DM was 50.7 per 100,000 person-years in the age group of 30–49 years, representing the great majority of new cases of DM [7]. The risk for developing T2DM increased markedly with age, being seven-fold higher in the age group of 40–49 years than in the age group of 30–34 years, irrespective of sex [7]. The incidence of T1DM has progressively increased in Italy with 3-4 times higher rates in Sardinia than in other parts of Italy [8, 9]. The Italian Health Service system estimates that 10 billion euros is the annual cost for the care of patients with DM, and these costs are increasing over time [10, 11].

## 2. Diabetic Retinopathy: Global and Italian Epidemiology Data 

Diabetic retinopathy (DR) is the leading cause of legal blindness among the working aged adults [12]. Nearly all the patients with T1DM and the majority of those with T2DM are affected by some form of DR after 20 years of disease duration and 50% may develop sight-threatening DR [13–15]. The main risk factors associated with an early onset and rapid evolution of DR are duration of DM, poor glycemic control, and presence of concomitant arterial hypertension [5]. The Wisconsin Epidemiologic Study of Diabetic Retinopathy (WESDR) reported that the incidence of diabetic macular edema (DME) is 29% in T1DM over a period of 25 years and 25.4% among those with T2DM requiring insulin [13, 14, 16]. A pooled analysis from 35 studies worldwide (from 1980 to 2008) evaluating more than 20000 people with DM reported an overall prevalence of any DR of 34.6% (95% confidence interval) (CI, 34.5–34.8), proliferative DR (PDR) of 6.96% (CI, 6.87–7.04), DME of 6.81% (CI, 6.74–6.89), and sight-threatening DR of 10.2% (CI, 10.1–10.3) [17].

In Italy, there are no national data about prevalence and incidence of legal blindness due to DR, and there is no national registry of patients with DM [11]. However, several studies reported the prevalence and incidence of DR from geographically limited population-based studies [18–20]. In one of these studies, 1321 patients with DM were examined for DR in the Veneto Region (northeast of Italy). DR prevalence was 26.2% (24.4% background DR and 1.8% PDR) as reported in 1991 [18]. The prevalence of DR was significantly related (*p* < 0.01) to the duration of DM (17.3% for <5 years; 60.8% for >20 years) [18]. In the province of Torino (northwest of Italy), DR was the second most common cause of bilateral blindness (13.1%) in 4549 residents who were certified blind between 1967 and 1991 [19]. Of the 6857 consecutive patients seen between 1992 and 2003, the prevalence of DR was 39% (19% mild nonproliferative DR (NPDR), 11% moderate NPDR, and more severe in the remaining cases) [21]. Furthermore, data collected by general practitioners and diabetes specialists in Italy reported in 1997 that 13% of patients with diabetes had PDR and 2% suffered from blindness [20].

In the province of Viterbo (located in the Lazio Region, Central Italy) in 2002, DR was the fourth most frequent cause of blindness accounting for 15% of cases [22]. When DM is diagnosed after 30 years of age, the prevalence of DR is about 20% after 5 years, 40–50% after 10 years, and >90% after 20 years of disease [19]. The cumulative incidence of DR ranged from 34% to 59% during a four-year period, depending on the age of patient and severity of disease [18, 19]. As a whole, DR was responsible for 13% of cases of severe visual impairment in Italy [18, 19].

Therefore, screening for DR remains crucial for early diagnosis of the disease and preventing blindness and is recommended in all patients with DM [23–25]. The* “Associazione Medici Diabetologi” (AMD)*,* “Società Oftalmologica Italiana*,*” “Società Italiana della Retina*,*” “Società Italiana di Diabetologia*,*”* and other organizations have jointly published a guideline for the screening, diagnosis, and treatment of DR in Italy, the* “Linee-Guida Retinopatia Diabetica”* [26].

However, screening for DR is delivered to only approximately half of all patients with DM (as reported in the United States), where the annual fundus examination was recommended as the annual screening for DR [27, 28]. As a consequence, the access to the treatment has been also limited for these patients. The use of retinal photography with an overall sensitivity of approximately 85% is considered currently the standard method to be used in a screening setting, especially as it allows for implementation of telemedicine programs [29–32].

## 3. Telemedicine Screening Programs in Italy

Data about the use of telemedicine for DR screening in Italy are very limited in the literature. Vujosevic et al. underlined the reliability of nonmydriatic techniques used in screening and grading settings and confirmed the importance of digital images over ophthalmoscopic examination in screening and grading of DR [32]. These authors evaluated 3 nonmydriatic field color fundus photos covering 45 degrees consisting in field 1 (central), centered on the macula; field 2 (nasal), centered on the nasal margin of the optic disc; and field 3 (temporal), centered superiorly and temporally from the macula and compared to just one central fundus color photo and to 7 standard stereoscopic 30-degree photos (ETDRS fields) in detecting referable DR, defined as severe NPDR and PDR and DME [32]. Sensitivity and specificity for detecting referable DR were 82% and 92% and 83% and 97% for referable DME for 3 nonmydriatic fields fundus photos and significantly lower (below the requested target of the British Diabetic Association necessary for an effective screening, set at 80%) for one field fundus photo [32, 33].

In Torino the use of nonmydriatic fundus photos in the screening program was introduced in 2000 [34]. The fundus photos were taken in the diabetes center by trained nurses or medical personnel and consist in 2 nonmydriatic 45-degree color fundus photos, one centered on the macula/central field and the other centered on the optic disc (nasal field) as proposed by EURODIAB procedure [34, 35]. Grading was performed by diabetes specialists, after specific training, according to the European Working Party recommendations [34, 36]. Patients were assessed at retinal photography and formally graded later. Feedback on referrals was by direct discussion with the consultant ophthalmologists working in the DR Centre. The authors evaluated the 6-year cumulative incidence of referable DR and reported 10.5% (95% CI, 9.4–11.8) [34]. Referable DR was considered in case of moderate NPDR or worse (preproliferative DR, PDR, photocoagulated DR, and advanced diabetic eye disease with or without macular involvement), equivalent to Early Treatment of Diabetic Retinopathy Study (ETDRS) level >35 [37], whereas patients with mild NPDR, equivalent to an ETDRS level ≤35, did not require referral and were given rescreening appointments. Retinopathy progressed within 3 years to referable severity in 6.9% (95% CI, 4.3–11.0) of patients with age at onset ≥30 years, who were on insulin treatment and had a known disease duration of 10 years or longer. The other patients, especially those with age at onset <30 years, on insulin and with a duration <10 years, progressed more slowly [34]. The authors concluded that screening can be repeated safely at 2-year intervals in any patient without DR [34].

The telematic screening program for DR in Padova area (northeast of Italy) was systematically implemented for those with DM in 2005 and since then a total of 17344 screening exams of 9347 patients with DM have been performed (data reported up to 2015). Color fundus photos of patients with DM are acquired in two remote diabetes centers by qualified staff (nurses or technicians) and thereafter sent by dedicated intranet link to the Reading Centre, at the Department of Ophthalmology of the University of Padova. Images are acquired with the use of nonmydriatic fundus cameras. The grading of images is performed by certified medical personnel and confirmed by the responsible medical retina specialist. In order to minimize errors, all evaluations of images are performed in double grading fashion and in case of discordance all adjudications are performed by the experienced grader. The final grading report with the results of DR grading is sent back electronically to the referring Diabetes Clinic. Grading of images is based on the International Diabetic Retinopathy and Macular Edema Severity Scale [38]. The National Guidelines for Screening of DR are adopted for determining the follow-up of patients [26]. Patients without DR or with mild NPDR are recommended a reevaluation within 12 months in the screening service, while patients with moderate NPDR are rescreened within 6–10 months and patients with severe NPDR or proliferative DR or with any maculopathy are referred to the DR Clinic for a complete ophthalmologic examination with possibility to perform optical coherence tomography and fluorescein angiography, if necessary. If grading is not certain or not possible due to poor quality of images, a recommendation to repeat either the screening examination or the ophthalmologic evaluation is given (in 1.3% of cases). From 2005 to 2015, the overall prevalence of DR in the city of Padova was 27.6% consisting in 12.5% mild NPDR, 11.3% moderate NPDR, 2.9% severe NPDR, and 0.9% proliferative DR (PDR)* (manuscript submitted)*. The overall prevalence of maculopathy was 5.7% consisting in 2.8% mild, 2.2% moderate, and 0.7% severe maculopathy. The 10-year incidence of sight-threating DR (STDR) was 0.6% in patients with no DR, 5.5% in patients with mild NPDR, and 21.1% in patients with moderate NPDR at the first examination. The 10-year incidence of maculopathy was 2.1% mild, 1.7% moderate, and 0.2% severe maculopathy in patients with no maculopathy at the first examination. When evaluating type and duration of DM together, patients were divided into low risk, medium risk, and high risk as follows: T2DM and duration lower than 10 years—low risk patients; T1DM and duration lower than 10 years—medium risk patients; and duration higher than 10 years and either T1DM or T2DM—high risk patients. The best sensitivity/specificity ratio (94.4%/32.4%) was found at 2.5 years for low risk patients with no DR at first examination. Therefore, the authors concluded that screening for DR can be safely repeated in a two-and-a-half-year period in those patients with diabetes who were deemed to be low risk diabetics. However, in case of presence of risk factors, a more frequent follow-up regime is warranted* (manuscript submitted)*.

Another pilot screening program was recently performed (2012) in Ponzano, a part of the Local Health Authorities of Veneto Region, Treviso (northeast Italy), with participation of a multidisciplinary group including general practitioners, diabetes experts, administrative staff, nurses, epidemiologists and ophthalmologists, and the reading centre [39]. This project aimed to assess the feasibility of a future larger application, in comparison with the “no prevention” strategy. Screening for DR was based on 3 nonmydriatic, 45° field, color fundus photos, obtained according to a previously validated technique [32]. All photographs were obtained by trained paramedical staff. All images were electronically transmitted to the reading centre and stored in an on-line secured database for the second step examination by certified and expert graders from the Reading Centre, University of Padova. Retinal images were graded for DR and DME according to the International Classification proposed by the American Academy of Ophthalmology [38]. When the quality of the images was “inadequate” for the clinical evaluation and when fundus photographs were graded as “positive,” these patients were referred for further ophthalmologic examination. “Positive” findings included retinal changes that required ophthalmologic management: moderate and severe NPDR, proliferative DR, DME, or any other retinal abnormality. A report with the results of the screening and the correct follow-up timetable for the “negative” screened population was sent to the patient's general practitioner within 1 month after the screening. The authors reported that a total of 498 patients with diabetes were identified among the larger sample and were invited for screening, with an attendance rate of approximately 80%. Of these, 115 patients (33.82%) were referred to an ophthalmologist, including patients with ungradable images and cases with any abnormal retinal findings, other than DR. Moreover, 9 cases required prompt treatment for either PDR or DME. Significant importance of screening program, also from an economic point of view, was found, leading to a substantial saving in comparison with the “no prevention” strategy, including the costs that avoid blindness, in terms of the validity of the intervention and the direct costs absorbed (efficiency) by the Regional Healthcare Service (Veneto Region) [39].

In Milano, a recent observational study reported a 1-year (2012-2013), single-center, remote evaluation of semiautomatic fundus photography for DR screening performed at the Endocrinology Unit, during routine systemic visits for patients with T2DM [40]. A total of 1281 adults with T2DM underwent fundus photos consisting in three 30-degree fields color fundus photos (central, nasal, and temporal) obtained before and after pupil dilation and thereafter assessed by 2 expert ophthalmologists who were blinded to the results of the slit-lamp fundus examination. After pupil dilation 240 patients (18.74%) had ungradable images; approximately two thirds of patients (823 patients) did not have DR, and 218 (17.01%) were diagnosed with DR. Consequently, a total of 458 (35.75%) patients (240 ungradable and 218 with DR) were referred to the ophthalmologist. The authors evaluated also the economic impact of the telematic screening and reported a significant cost saving compared to slit-lamp fundus examination (the evaluated costs included reading centre staff evaluating images, fundus camera, and the cost of the standard funduscopic examination) [40].

Although initially annual screening for DR was recommended by many professional societies and was practiced in many countries, currently there is an increasing evidence of cost-effectiveness studies that suggest that screening could be safely extended beyond one year in patients with T2DM at low risk of progression to DR (considered to be patients with well controlled DM on dietary treatment, with low HbA1c and no DR), without increased risk of vision loss [41]. Biennial screening showed long safety record in Iceland and Sweden [42, 43]. Moreover, adopting biennial screening approach, a reduction in approximately 25% of screening costs can be obtained without increased risk to the patient [44]. Two recent studies reported cost-effectiveness of adopting risk-stratified approaches to extended screening intervals in the national DR screening programs in England and Scotland [45, 46]. Scanlon et al. conducted a modelling study and reported that for patients without DR on two consecutive screening examinations the adoption of 2-year screening intervals would save on average 225000 pounds per QUALY (quality-adjusted life years) lost compared with annual screening in England [45]. Scotland et al. reported similar results for patients with T2DM and lower increment in cost-effectiveness ratios for patients with T1DM (85000 pounds) per QUALY gained [46].

## 4. Conclusions

DR is a relevant and significant complication of DM and affects a large number of patients, with significant costs for the Health System. Prevention of DR through reducing risk factors and screening (early diagnosis) results in preventing visual impairment. Telematic screening for DR has been implemented with success in several local health entities in Italy, demonstrating good interdisciplinary collaboration and patient satisfaction. Moreover, with recent reports [45–49] on possibility to effectively increase screening intervals in patients with no DR and at low risk for developing sight-threating DR, the screening program becomes even more cost-effective procedure with appropriate use of resources and safe care delivered for patients. The preliminary data already present in the literature together with already available local experience in DR screening may become a basis for developing a national screening program.

## References

[1] Wild S., Roglic G., Green A., Sicree R., King H. (2004). Global prevalence of diabetes: estimates for the year 2000 and projections for 2030. *Diabetes Care*.

[2] Osservatorio ARNO Diabete Il profilo assistenziale della popolazione con diabete. Rapporto 2015; XXIII. http://osservatorioarno.cineca.org.

[3] Associazione Medici Diabetologi Annali AMD. http://www.aemmedi.it/pages/annali_amd/.

[4] Istituto Nazionale di Statistica (2012). *Annuario Statistico Italiano 2012*.

[5] Associazione Medici Diabetologi (2016). *Standard Italiani per la Cura del Diabete Mellito 2016*.

[6] Bonora E., Kiechl S., Willeit J. (2004). Population-based incidence rates and risk factors for type 2 diabetes in white individuals: the Bruneck study. *Diabetes*.

[7] Bruno G., Runzo C., Cavallo-Perin P. (2005). Incidence of type 1 and type 2 diabetes in adults aged 30–49 years: the population-based registry in the province of Turin, Italy. *Diabetes Care*.

[8] Cherubini V., Mascioli G., Carle F. (2004). L'incidenza del diabete mellito tipo 1 nell’età infantile: lo studio RIDI. *Il Diabete*.

[9] Casu A., Songini M. (2004). Diabete mellito tipo 1 nell’adulto. In Il diabete mellito in Italia—parte prima: epidemiologia. *Il Diabete*.

[10] De Berardis G., D'Ettorre A., Graziano G. (2012). The burden of hospitalization related to diabetes mellitus: a population-based study. *Nutrition, Metabolism and Cardiovascular Diseases*.

[11] Disoteo O., Grimaldi F., Papini E. (2015). State-of-the-art review on diabetes care in Italy. *Annals of Global Health*.

[12] Klein B. E. K. (2007). Overview of epidemiologic studies of diabetic retinopathy. *Ophthalmic Epidemiology*.

[13] Klein R., Klein B. E. K., Moss S. E. (1984). The wisconsin epidemiologic study of diabetic retinopathy. II. Prevalence and risk of diabetic retinopathy when age at diagnosis is less than 30 years. *Archives of Ophthalmology*.

[14] Klein R., Klein B. E. K., Moss S. E. (1984). The wisconsin epidemiologic study of diabetic retinopathy. III. Prevalence and risk of diabetic retinopathy when age at diagnosis is 30 or more years. *Archives of Ophthalmology*.

[15] Klein R., Klein B. E. K., Moss S. E. (2003). Retinal vascular abnormalities in persons with type 1 diabetes: the Wisconsin Epidemiologic Study of Diabetic Retinopathy: XVIII. *Ophthalmology*.

[16] Klein R., Klein B. E. K., Moss S. E., Cruickshanks K. J. (1998). The wisconsin epidemiologic study of diabetic retinopathy: XVII. The 14- year incidence and progression of diabetic retinopathy and associated risk factors in type 1 diabetes. *Ophthalmology*.

[17] Yau J. W. Y., Rogers S. L., Kawasaki R. (2012). Global prevalence and major risk factors of diabetic retinopathy. *Diabetes Care*.

[18] Segato T., Midena E., Grigoletto F. (1991). The epidemiology and prevalence of diabetic retinopathy in the Veneto region of North East Italy. Veneto Group for Diabetic Retinopathy. *Diabetic Medicine*.

[19] Porta M., Tomalino M. G., Santoro M. G. (1995). Diabetic retinopathy as a cause of blindness in the province of Turin, North-west Italy, in 1967–1991. *Diabetic Medicine*.

[20] Nicolucci A., Scorpiglione N., Belfiglio M. (1997). Patterns of care an Italian diabetic population. The Italian Study Group for the Implementation of the St Vincent Declaration, Società Italiana di Diabetologia, Associazione Medici Diabetologi. *Diabetic Medicine*.

[21] Porta M., Taulaigo A. V. (2014). The changing role of the endocrinologist in the care of patients with diabetic retinopathy. *Endocrine*.

[22] Cruciani F., Abdolrahimzadeh S., Vicari A., Amore F. M., Di Pillo S., Mazzeo L. (2010). Causes of blind certification in an Italian province and comparison with other European countries. *La Clinica Terapeutica*.

[23] Garvican L., Clowes J., Gillow T. (2000). Preservation of sight in diabetes: developing a national risk reduction programme. *Diabetic Medicine*.

[24] American Diabetes Association (1998). Diabetic retinopathy. *Diabetes Care*.

[25] Royal College of Ophthalmologists (1997). *Guidelines for Diabetic Retinopathy*.

[26] Linee-guida per lo screening, la diagnostica e il trattamento della retinopatia diabetica in Italia, 2015, http://docplayer.it/4760396-Linee-guida-per-lo-screening-la-diagnostica-e-il-trattamento-della-retinopatia-diabetica-in-italia.html

[27] McGlynn E. A., Asch S. M., Adams J. (2003). The quality of health care delivered to adults in the United States. *The New England Journal of Medicine*.

[28] Lee P. P., Feldman Z. W., Ostermann J., Brown D. S., Sloan F. A. (2003). Longitudinal rates of annual eye examinations of persons with diabetes and chronic eye diseases. *Ophthalmology*.

[29] Pugh J. A., Jacobson J. M., Van Heuven W. A. J. (1993). Screening for diabetic retinopathy: the wide-angle retinal camera. *Diabetes Care*.

[30] Harding S. P., Broadbent D. M., Neoh C., White M. C., Vora J. (1995). Sensitivity and specificity of photography and direct ophthalmoscopy in screening for sight threatening eye disease: the Liverpool diabetic eye study. *British Medical Journal*.

[31] Telemedicine A. (2005). Program for diabetic retinopathy in a veterans affairs medical center—the joslin vision network eye health care model. *American Journal of Ophthalmology*.

[32] Vujosevic S., Benetti E., Massignan F. (2009). Screening for diabetic retinopathy: 1 and 3 nonmydriatic 45-degree digital fundus photographs vs 7 standard early treatment diabetic retinopathy study fields. *American Journal of Ophthalmology*.

[33] British Diabetic Association (1997). *Retinal Photographic Screening for Diabetic Eye Disease. A British Diabetic Association Report*.

[34] Porta M., Maurino M., Severini S. (2013). Clinical characteristics influence screening intervals for diabetic retinopathy. *Diabetologia*.

[35] Aldington S. J., Kohner E. M., Meuer S., Klein R., Sjølie A. K. (1995). Methodology for retinal photography and assessment of diabetic retinopathy: the EURODIAB IDDM complications study. *Diabetologia*.

[36] Kohner E. M. (1991). A protocol for screening for diabetic retinopathy in Europe. *Diabetic Medicine*.

[37] Early Treatment Diabetic Retinopathy Study Research Group (1991). Grading diabetic retinopathy from stereoscopic colour fundus photographs—an extension of the modified Airlie House classification. ETDRS report no 10. *Ophthalmology*.

[38] Wilkinson C. P., Ferris F. L., Klein R. E. (2003). Proposed international clinical diabetic retinopathy and diabetic macular edema disease severity scales. *Ophthalmology*.

[39] Scarpa G., Urban F., Vujosevic S. (2016). The nonmydriatic fundus camera in diabetic retinopathy screening: a cost-effective study with evaluation for future large-scale application. *Journal of Ophthalmology*.

[40] Invernizzi A., Bevilacqua M. T., Cozzi M. (2016). Diabetic retinopathy screening: the first telemedical approach in an Italian hospital. *European Journal of Ophthalmology*.

[41] Taylor-Phillips S., Mistry H., Leslie R. (2016). Extending the diabetic retinopathy screening interval beyond 1 year: systematic review. *British Journal of Ophthalmology*.

[42] Ólafsdóttir E., Stefánsson E. (2007). Biennial eye screening in patients with diabetes without retinopathy: 10-year experience. *British Journal of Ophthalmology*.

[43] Lund S. H., Aspelund T., Kirby P. (2016). Individualised risk assessment for diabetic retinopathy and optimisation of screening intervals: a scientific approach to reducing healthcare costs. *British Journal of Ophthalmology*.

[44] Javitt J. C., Aiello L. P., Chiang Y., Ferris F. L., Canner J. K., Greenfield S. (1994). Preventive eye care in people with diabetes is cost-saving to the federal government. Implications for health-care reform. *Diabetes Care*.

[45] Scanlon P. H., Aldington S. J., Leal J. (2015). Development of a cost-effectiveness model for optimisation of the screening interval in diabetic retinopathy screening. *Health Technology Assessment*.

[46] Scotland G., McKeigue P., Philip S. (2016). Modelling the cost-effectiveness of adopting risk-stratified approaches to extended screening intervals in the national diabetic retinopathy screening programme in Scotland. *Diabetic Medicine*.

[47] Agardh E., Tababat-Khani P. (2011). Adopting 3-year screening intervals for sight-threatening retinal vascular lesions in type 2 diabetic subjects without retinopathy. *Diabetes Care*.

[48] Aspelund T., Thornórisdóttir O., Olafsdottir E. (2011). Individual risk assessment and information technology to optimise screening frequency for diabetic retinopathy. *Diabetologia*.

[49] Looker H. C., Nyangoma S. O., Cromie D. T. (2013). Predicted impact of extending the screening interval for diabetic retinopathy: the Scottish Diabetic Retinopathy Screening programme. *Diabetologia*.

